# Determining the anti-coagulant-independent anti-cancer effects of heparin

**DOI:** 10.1038/sj.bjc.6605808

**Published:** 2010-07-20

**Authors:** V Solari, E C Jesudason, J E Turnbull, E A Yates

**Affiliations:** 1 Centre for Glycobiology, School of Biological Sciences, University of Liverpool, Crown Street, Liverpool L69 3BX, UK; 2 Saban Research Institute, Childrens Hospital Los Angeles, University of Southern California (USC), 4650 Sunset Boulevard, Los Angeles, CA 90027, USA

**Sir,**

We enjoyed the recent articles on cancer-associated thrombosis and wish to comment on the attempts to explain the beneficial effects of heparin in cancer (Noble and Pasi, 2010; Kakkar and Macbeth, 2010).

Cited evidence indicates that the survival benefit of heparin in cancer is unexplained by venous thromboembolism (VTE) prophylaxis alone; unlike warfarin, heparin improves survival in cancer patients without VTE (Zielinski and Hejna, 2000; Cunningham *et al*, 2009). Alternative explanations independent of VTE prophylaxis include regulation of tissue factor and FVIIa with the TF–VIIa complex (Boccaccio and Comoglio, 2005; Rickles and Falanga, 2009), inhibition of cancer micrometastasis by blocking platelet and cancer cell aggregation (Borsig *et al*, 2001), and disruption of host wound-healing responses (including clotting and fibrin deposition) proposed to facilitate construction of tumour microenvironment (Dvorak, 1986). Although these explanations do not require VTE prophylaxis, they still relate to heparin's clotting-related effects.

Heparin is a multifunctional, highly sulphated form of heparan sulphate (HS), a member of the glycosaminoglycan family. Given the vast interactome of HS and related heparins (Ori *et al*, 2008), it is unsurprising that heparin's benefit in cancer cannot be linked solely to its anti-coagulant activity (Fuster and Esko, 2005; Escobar Galvis *et al*, 2007). Estimated to carry far more information than nucleic acids, the HS family are ubiquitous, varied and highly complex regulators of normal organogenesis (e.g., through growth factor modulation) with pleiotropic biological effects unrelated to anti-coagulation (Bishop and Schuksz Esko, 2007). Likewise, heparin has diverse, structure-related biological properties, only one of which is anti-coagulation and this can be manipulated through judicious structural modification to generate low-molecular-weight derivatives with improved selectivity (Norrby, 1993). Clinicians glimpsed heparin's diversity during replacement of unfractionated heparins with fractionated low-molecular-weight alternatives (although the focus remained anti-coagulation).

If they are entirely anti-coagulant related, further exploitation of heparin's benefits in cancer is inevitably limited by haemorrhage risk. However, by separating anti-coagulant from other bioactivities, ‘engineered’ (selectively modified) heparins provide the means to test whether heparins mediate their benefits in cancer independently of coagulation effects, as has been achieved for other biological activities such as inhibition of *β*-secretase or blood cell rosetting in malaria (Patey *et al*, 2006; Yates *et al*, 1996; Skidmore *et al*, 2008). Such developments offer the prospect of screening and developing a broad new class of anti-cancer heparins that can be used at optimum anti-tumour levels while retaining ameliorated anti-thrombotic activity.
